# Spindle‐Shaped Ni‐Fe‐Layered Double Hydroxide: Effect of Etching Time on Flexible Energy Storage

**DOI:** 10.1002/smll.202409959

**Published:** 2025-01-10

**Authors:** Keerthi M. Nair, Sindhya Ajith, Febin Paul, Sreedhanya Pallilavalappil, Nishanth Thomas, Steven J. Hinder, Libu Manjakkal, Suresh C. Pillai

**Affiliations:** ^1^ Nanotechnology and Bio‐Engineering Research Group Atlantic Technological University ATU Sligo Ash Lane Sligo F91 YW50 Ireland; ^2^ Health and Biomedical (HEAL) Research Centre Atlantic Technological University ATU Sligo Ash Lane Sligo F91 YW50 Ireland; ^3^ School of Computing and Engineering & the Built Environment Edinburgh Napier University Merchiston Campus Edinburgh EH10 5DT UK; ^4^ The Surface Analysis Laboratory Faculty of Engineering and Physical Sciences University of Surrey Guildford Surrey GU2 7XH UK

**Keywords:** flexible energy storage, layered hydroxide, metal organic framework, NiFe‐LDH, supercapacitor

## Abstract

The rising demand for efficient energy storage in flexible electronics is driving the search for materials that are well‐suited for the fabrication of these devices. Layered Double Hydroxides (LDHs) stand out as a remarkable material with a layered structure that embodies exceptional electrochemical properties. In this study, both double‐shelled and single‐shelled NiFe‐Layered Double Hydroxide (LDH) particles are prepared using spindle‐shaped MIL‐101(Fe) as the template. These NiFe‐LDH particles are then utilized to develop a flexible energy storage device. Transmission electron microscopy(TEM) analysis revealed that the as‐synthesized NiFe‐LDH particles transformed into hollow single‐shells from a double‐shelled structure as the aging time increased, which significantly influenced the electrochemical performances. Despite the decreasing specific capacitance and energy density with longer etching times, the sample etched for 2 h (NiFe‐LDH 2h) demonstrated the highest capacitance of 9.24 mF·cm⁻^2^ and an energy density of 0.46 µW·h·cm⁻^2^, highlighting its promising performance for energy storage applications. X‐ray photoelectron spectroscopy (XPS) analysis revealed the highest Ni^2+^: Ni^3+^ ratio, and Fe: Ni ratio for NiFe‐ LDH 2h samples, which further influences the energy storage properties. The ability to maintain the high performance of these materials across different bending angles further emphasizes its versatility and relevance in emerging flexible electronics markets.

## Graphical abstract

Herein, double‐shelled, and single‐shelled NiFe‐LDH particles are obtained using the octahedral spindle‐shaped MIL‐101(Fe) as the template. The as‐synthesized NiFe‐LDH particles are observed to transform into hollow single‐shells from a double‐shelled structure as the aging time increased. The energy‐storing performances of these NiFe‐LDH are investigated through the design of flexible supercapacitors (FSCs).

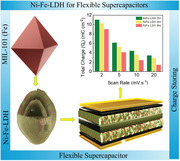

## 1 Introduction

Over the past few decades, there has been a growing interest in ultrathin 2D nanomaterials as catalysts, owing to their numerous active defect sites, manageable electronic structures, and impressive capacity for charge separation.^[^ ^1^ ^]^ Among these catalysts, LDHs have emerged as promising candidates, particularly in the context of energy storage in electrochemical capacitor applications.^[^ ^2^ ^]^ LDHs are a class of versatile materials with a layered structure, with a general formula of [M^II^_1−x_M^III^_x_ (OH)_2_]^x+^[A^n −^_x/n_·yH_2_O]^x−^, where M(II) and M(III) are divalent and trivalent metal cations, respectively, and A^n−^ is an n‐valent anion, respectively.^[^ ^3^ ^]^ It is widely recognized that the effectiveness of a material and its performance in electrochemical applications is greatly influenced by its morphology.^[^ ^4^ ^]^ However, the conventional synthesis techniques for LDHs offer limited control over key factors like morphology, particle size, and surface area, leading to particle coagulation and limiting mass transfer.^[^ ^5^ ^]^ Therefore, selecting an appropriate template for fabricating nanostructures, with desired morphologies and properties would be a key to achieving higher catalytic and electrochemical energy‐storing performances.^[^ ^6^ ^]^ Metal‐organic frameworks (MOFs), renowned for their crystalline structures composed of metal nodes interconnected by organic linkers, offer a vast playground for constructing precisely engineered architectures. The inherent porosity of MOFs creates confined spaces, effectively serving as molds or scaffolds for the nucleation and crystallization of LDHs.^[^ ^7^ ^]^

Recently, studies have been conducted on the MOF‐directed growth of LDHs, focusing on applications such as electrocatalysis and energy storage systems.^[^ ^6^, ^8^ ^]^ In one of these studies, NiCo‐LDHs, derived from the controlled hydrolysis of MOFs, demonstrated a remarkable electrochemical performance. These bimetallic hydroxides exhibited a specific capacitance of ≈1652 F. g^−1^, which could be attributed to the multiple oxidation states of the materials.^[^ ^9^ ^]^ However, the rate of etching and the inheritance of the morphology of the MOF template are highly influenced by factors such as temperature, duration of the hydrolysis/etching, concentration of the metal precursors, and solvent ratios.^[^ ^7^, ^10^ ^]^ It was observed that compared to single‐shelled hollow configurations, the hollow catalysts with a multi‐shelled design demonstrate notable advantages, including an enlarged surface area and more effective utilization of the internal volume^[^ ^10^, ^11^ ^]^ which further enhances the electrochemical performances. This multi‐shelled design of MOF‐derived LDHs can be achieved by tailoring the morphology and the number of shells through controlled manipulation of hydrolysis/etching time, as well as by regulating template etching and metal precipitation durations. A recent study reported the synthesis of NiCo‐LDHs through the in situ etching of a Ni‐MOF template over varying hydrolysis durations. It was observed that the nickel‐cobalt LDH (NiCo‐LDH)/ten samples, synthesized after a 10‐h reaction, featured a high surface area and large LDH sheets on microspheres. This structure facilitated rapid electrolyte ion transport, resulting in a maximum specific capacity of 1272 F. g^−1^ at a current density of 2 A. g^−1^ in a supercapacitor (SC).^[^ ^12^ ^]^

**Figure 1:**
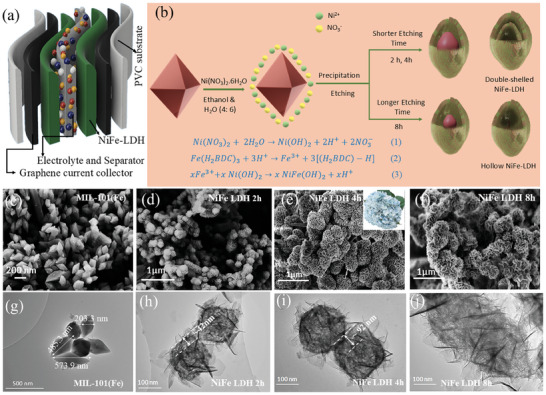
a) Schematic representation of NiFe‐LDH‐based flexible SC. b) The formation illustration of the NiFe‐LDH nanocages by simultaneous precipitation and etching. c–f) FE‐SEM images of c) MIL‐101(Fe), d) NiFe‐LDH 2 h, e) NiFe‐LDH 4 h, and f) NiFe‐LDH 8 h. g) TEM images of MIL‐101(Fe), h–j) TEM images of (h) NiFe‐LDH 2 h (4000x magnification), i) NiFe‐LDH 4 h (4000x magnification) and j) NiFe‐LDH 8 h (4000x magnification).

In this work, double‐shelled, and single‐shelled NiFe‐LDH particles were obtained using the octahedral spindle‐shaped MIL‐101(Fe) MOFs as the template. LDH samples were prepared by varying the etching times (2, 4, and 8 h), and the energy‐storing performances of these NiFe‐LDH were investigated through the design of FSCs, as shown in **Figure** **1**a. As the aging time increased, the as‐synthesized NiFe‐LDH particles were observed transforming into hollow single‐shells from a double‐shell structure. NiFe LDH is a remarkable material with a layered structure that embodies exceptional electrochemical properties. Like other LDH, its structure comprises positively charged brucite‐like layers interspersed with divalent metal ions, primarily nickel and iron, occupying octahedral sites.^[^ ^13^ ^]^ These layers, held together by weak van der Waals forces, facilitate facile intercalation and ion exchange, contributing to its high specific surface area.^[^ ^14^ ^]^ This high surface area, coupled with the redox activity of nickel and iron ions within the structure, engenders excellent electrochemical performance. NiFe LDH offers tunability in composition, allowing for fine adjustments to tailor its properties for specific applications.^[^ ^15^ ^]^ In the realm of energy storage, NiFe LDH demonstrates remarkable capacitance characteristics. Its ability to store charge via both double‐layer capacitance, through reversible adsorption of ions at the electrode‐electrolyte interface, and pseudo capacitance, owing to reversible redox reactions of nickel and iron ions, results in high specific capacitance values. This amalgamation of structural integrity and electrochemical prowess positions NiFe LDH as a promising candidate for advanced energy storage devices, particularly in SCs, where its high capacitance performance could revolutionize energy storage technology.^[^ ^16^ ^]^ For the fabrication, cellulose cloth was used as a separator and 6 m KOH as an electrolyte. Three flexible SCs were prepared, namely FSC1 (NiFe‐LDH 2 h), FSC2 (NiFe‐LDH 4 h) and FSC3 (NiFe‐LDH 8 h). The electrochemical characterization through the 2‐electrode measurement of NiFe LDH FSC samples with varying etching times (2, 4, and 8 h) revealed insightful trends regarding their performance. The cyclic voltammetry (CV) and galvanostatic charging discharging (GCD) analyses suggest pseudo‐capacitive behavior, with quasi‐rectangular CV curves and non‐triangular GCD curves, indicating the coexistence of redox reactions and electric double‐layer formation. Electrochemical impedance spectroscopy (EIS) analysis measurements unveil the electrode‐electrolyte interaction of the FSCs. Among the fabricated FSCs, the 2 h etching time sample exhibited the highest capacitance (9.24 mF.cm^−^^2^) and energy density (0.46 µW.h. cm^−^^2^), showcasing its promising performance for energy storage applications.

## 2 Results and Discussion

Figure 1b represents the major reaction mechanism responsible for the conversion of MIL‐101(Fe) to NiFe‐LDH through the hydrolysis of Ni(NO_3_)_2_.6H_2_O precursor. Here, when Ni(NO_3_)_2_·6H_2_O is added to the solution containing MIL‐101(Fe), the hydrolysis of the metal nitrate generates protons that react with the surface of MIL‐101(Fe). The generated protons lead to the breakage of the coordinate bond between the 1,4 benzene dicarboxylic acid (H_2_BDC) linkers and the Fe^3+^ sites in MIL‐101(Fe), releasing Fe^3+^ ions into the solution. This free Fe^3+^ could then combine with the hydrolyzed Ni ions via coprecipitation, depositing on the surface of MIL‐101(Fe) to form NiFe‐LDH.^[^ ^4^, ^7^, ^10^ ^]^ The morphology formation is further confirmed through the field emission scanning electron microscopy (FE‐SEM) and TEM analysis.

FE‐SEM images of MIL‐101(Fe) exhibit octahedral crystals with polished (glossy) surfaces with an equatorial diameter of 200−300 nm and a length of 400−600 nm as shown in Figure 1c. Table TS1 (Supporting Information) summarizes the particle size of MIL‐101(Fe) analyzed using ImageJ. Figure 1d–f indicates the formation of the LDH nanosheets on the surface of the MIL‐101(Fe). It can be observed that with the increase in the reaction time from 2 to 8 h, densely stacked sheets of NiFe‐LDH were formed. This can be attributed to the fact that longer reaction/etching time aids in higher etching and continuous deposition of metal ions resulting in the generation of thicker LDH shells on the MOF surface.^[^ ^10^ ^]^ However, the higher number of flakes leads to dense aggregation forming an extremely interconnected structure, showing a hydrangea‐like morphology.^[^ ^8^ ^]^

To further investigate the details of the formation of LDH layers on the MOF template, TEM analysis of all three NiFe‐LDHs synthesized at different etching times was conducted. Figure 1g shows the TEM image of MIL‐101(Fe) exhibiting even octahedral spindles with high purity and uniformity with an equatorial diameter of 200–300 nm and length 400–600 nm. Figure 1h confirms that the spindle morphology of MIL‐101(Fe) was inherited by the NiFe‐LDH, irrespective of the aging time. NiFe‐LDH 2 h and NiFe‐LDH 4 h show a double‐shelled structure, with MIL‐101(Fe) in its core and the LDH sheets starting to form on the outside shell. It also indicates that the inner core comprises of ultrathin sheets, with a central void of ≈42 nm between the two (Table TS2, Supporting Information).^[^ ^10^ ^]^ Figure 1i shows the TEM image of NiFe‐LDH 4 h, indicating that the central void space starts increasing with the prolonged reaction time, eventually leading to the formation of a complete hollow structure, NiFe‐LDH 8 h, after 8 h of etching time, as shown in Figure 1j. Figure S1 (Supporting Information) demonstrates a gradual transition from the double‐shelled to hollow structure and the extremely interconnected structure of NiFe‐LDH 8 h. Figure S2 (Supporting Information) shows the high resolution TEM (HRTEM) image of NiFe LDH‐2 h demonstrating an interlayer distance (d‐spacing) of ≈0.238 nm, indexed to the (012) plane of NiFe LDH. The selected area electron diffraction (SAED) patterns further evidence that most particles orient in the (012) plane, which is consistent with previous reports.^[^ ^17^ ^]^

**Figure 2:**
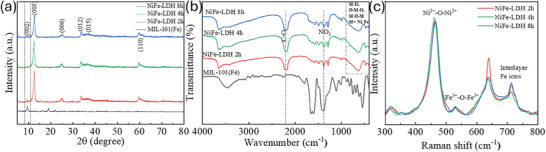
a) XRD patterns of MIL‐101(Fe) and the as‐synthesized NiFe‐LDH. b) FTIR spectra of MIL‐101(Fe), NiFe‐LDH 2 h, NiFe‐LDH 4 h, and NiFe‐LDH 8 h. c) Raman spectra of NiFe‐LDH 2 h, NiFe‐LDH 4 h and NiFe‐LDH 8 h.

The X‐ray diffraction (XRD) spectra of MIL‐101(Fe) and the NiFe‐LDH prepared at different times, indicating the formation of well‐crystallized particles are shown in **Figure** **2**a. The diffraction peaks at 9.1°, 18.5°, and 21.8°, for MIL‐101(Fe) matched well with the existing reports, indicating the formation of high‐purity crystals.^[^ ^18^ ^]^ It can be observed from the XRD spectra that with the increase in etching time, the intensity of the peak at 9.1°, corresponding to MIL‐101 (Fe) reduces gradually, indicating a higher degree of etching and complete transformation of MOF to LDH. No prominent impurity peaks were observed for any of the LDH samples, and the peaks at 11.6°, 23.2°, 34.5°, and 59.9° correspond to (003), (006), (012), and (110), respectively. (ICDD card no. 01‐082‐8040).^[^ ^19^ ^]^ Table TS3 (Supporting Information) shows interlayer spacing and crystal size values for different etching times. The fourier transform infrared (FTIR)X spectra of MIL‐101(Fe), NiFe‐LDH 2 h, NiFe‐LDH 4 h, and NiFe‐LDH 8 h are shown in Figure 2b. The intense peaks at 1392 and 1600 cm^−1^ represent the symmetric and asymmetric vibrations of carboxyl groups (─COO─) of the organic linkers in MIL‐101(Fe).^[^ ^18^ ^]^ The band at 553 cm^−1^ is assigned to the stretching vibration of Fe─O, proving the presence of iron‐oxo clusters in MIL‐101(Fe). However, irrespective of the aging time, all three NiFe‐LDH samples showed similar spectral patterns, consistent with the previous reports.^[^ ^20^ ^]^ The peaks at 3440 cm^−1^ correspond to the stretching mode of the hydroxyl groups in the surface‐absorbed water molecules. The distinct sharp peak ≈1377 cm^−1^ is indicative of the vibration mode of the interlayer nitrate ions and the peaks from 400 to 800 cm^−1^ are associated with the lattice vibration modes of M−O−M, M−O, and O−M−O, where M stands for Ni and Fe.^[^ ^21^ ^]^ Figure 2c shows the Raman spectra of NiFe‐LDH 2 h, NiFe‐LDH 4 h, and NiFe‐LDH 8 h. The major peaks ≈460 and 530 cm^−1^ are assigned to the Fe^3+^/Ni^2+^−O−Ni^2+^ and Fe^3+^−O−Fe^3+^modes.^[^ ^22^ ^]^ It is observed that the peak at 460 cm^−1^ shows a red shift for the 4 h sample, indicating a modification in the local structure of Ni‐O bonds, such as a lattice disorder or interlayer spacing within the LDH sheets. The peaks at 640 and 730 cm^−1^ represent the metal‐oxygen symmetric stretching vibrations, such as Ni‐O and Fe‐O stretching vibrations as well as Ni‐O‐Fe interactions, within the lattice.^[^ ^23^ ^]^

**Figure 3:**
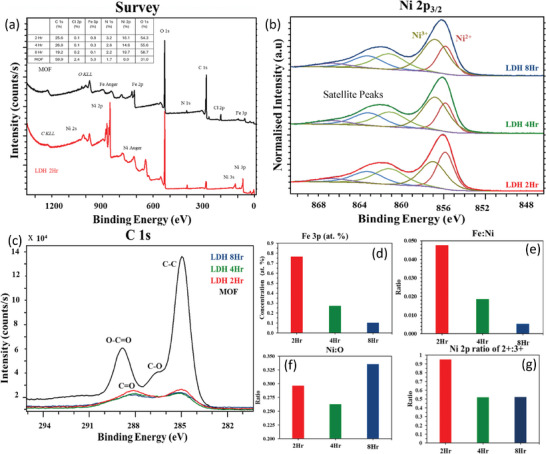
a) XPS survey spectra and high‐resolution spectra of b) Ni 2p_3/2_, c) C 1s, d) change in the atomic percentage of Fe with the aging time, e) ratio of Fe: Ni, f) Ni: O ratio and g) ratio of Ni^2+^ to Ni^3+^ with the aging time.

XPS analysis was conducted to ascertain the elemental composition and oxidation states of the as‐synthesized NiFe‐LDH samples as shown in **Figure** **3**. Figure 3a shows the survey spectra of both MIL‐101(Fe) and Ni‐Fe LDH indicating the presence of Fe 2p and Fe 3p in MIL‐101(Fe) and Ni 2p, Ni 3p, and Fe 2p in NiFe‐LDH samples, respectively. The survey spectra of all three samples are shown in Figure S3 (Supporting Information). Figure 3b presents the high‐resolution spectra of Ni 2p, with Ni 2p_3/2_ peak fitted to include its constituent components. The component peaks observed at 855.9 and 857.1 eV correspond to the Ni^2+^ and Ni^3+^ oxidation states respectively, while the peaks at 861.5, 863.7, and 867.1 eV are attributed to Ni2p_3/2_ satellite peaks. The ratio of Ni^2+^ to Ni^3+^ gradually reduces with the increase in the aging time, with the greatest concentration of Ni^2+^ observed in the NiFe‐LDH 2 h sample. The XPS spectra of C1s indicate the presence of intense O─C═O, C─O, and C─C peaks for MIL‐101(Fe), which eventually disappear in the generated NiFe‐LDH sample. This suggests that during the reaction, the organic linkers present in MIL‐101(Fe) break and release the free metal ions, aiding the formation of the LDH (Figure 3c).^[^ ^4^, ^7^ ^]^ The predominant oxidation state of Fe species in the samples is found to be mainly +3.^[^ ^24^ ^]^ Distinct peaks for Fe species were impossible to fit since there was a substantial convolution between Fe 2p and Ni LMM Auger peaks.

Figure 3d,e suggests that with the increase in the aging time, the ratio of Fe to Ni and the total Fe content gradually reduces. This can be attributed to the total consumption of all the free Fe^3+^ ions to form NiFe‐LDH as the aging time increases. Consequently, the hydrolyzed Ni ions remain in the solution, indicating an increase in the concentration of Ni. It was also observed that the ratio of Ni to O shows a similar trend as observed in the Raman spectra, indicating a local structural change of Ni‐O bonds, such as a lattice disorder or interlayer spacing within the LDH sheets (Figure 3f). Figure 3g indicates that the ratio of Ni^2+^: Ni^3+^ decreases with the increase in aging time. The generation of Ni^3+^ ions may result from the oxidation of Ni^2+^ facilitated by oxidizing agents like NO_3_^−^ and O_2,_ present in the reaction mixture. As the aging progresses, a higher rate of oxidation could contribute to a decreased ratio of Ni^2+^: Ni^3+^, with NiFe‐LDH 2 h having the highest concentration of Ni^2+^.^[^ ^18^, ^25^ ^]^

In addition, wettability is one of the important factors for an energy storage electrode and is correlated with the porosity of the electrode. A more porous electrode generally provides better wettability. The contact angle measured for the NiFe‐LDH 2 h sample (used in FSC‐1) is ≈13 degrees, 30 s after applying the electrolyte, as shown in Figure S4a,b (Supporting Information). The high porosity and highly interconnected structure of the NiFe‐LDH 2 h (FSC‐1 electrode), enhance its wettability, leading to effective absorption of the electrolyte. This improved wettability and porosity contribute to better electrochemical performance by facilitating ion penetration into the electrode's core structure. Additionally, XRD analysis revealed that samples derived from shorter etching times exhibited improved crystallinity. The presence of Fe and Ni ions further enhances the electrode's electronic conductivity, leading to better performance in energy storage. Consequently, the NiFe‐LDH 2 h electrode demonstrated an electrical conductivity of 789.01 S·m⁻¹, contributing to its superior performance in energy storage.

**Figure 4:**
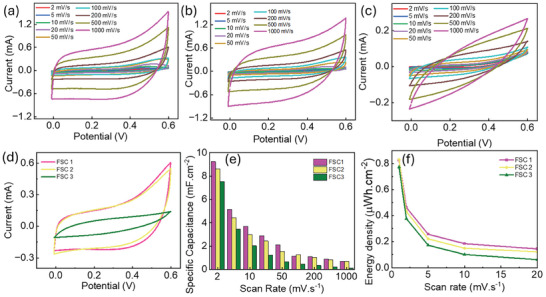
a–c) CV curve for FSC 1 (NiFe‐LDH 2 h), FSC2 (NiFe‐LDH 4 h), FSC3 (NiFe‐LDH 8 h) for 2–1000 mV.s^−1^. d) Comparison of CV curve for the devices e) the measured specific capacitance variation of the FSC with scan rate f) energy density variation with scan rate.

The electrochemical reactions of the NiFe‐LDH samples were investigated through CV analysis with two electrode symmetric configurations at a voltage window ranging from 0 to 0.6 V, with scan rates varying from 2 to 1000 mV.s^−1^, as illustrated in **Figure** **4**a–c for FSC1‐FSC3. The CV curve shows that as the scan rate increases, the peak current also rises, indicating a faster ion reaction. The observed curve shape suggests that both redox reactions and electric double layer formation (EDLC) are occurring, reflecting a pseudo‐capacitive behavior of the FSCs. Figure 4d compares the CV curves of different FSCs, with FSC1 showing a larger area under the curve than the other devices, indicating its superior performance. No obvious redox peaks were observed in the CV curve, which can be attributed to the CV analysis being conducted in a full symmetric cell, where charging and discharging occur at a pseudo‐constant rate throughout the entire voltametric cycles.^[^ ^26^ ^]^ An aqueous electrolyte such as KOH was used in this study, and the CV analysis was carried out within a low‐voltage window. A similar observation has been reported for other pseudocapacitive materials,^[^ ^27^ ^]^ including Fe–Ni_3_Co_2_ LDH,^[^ ^6^ ^]^ Co‐Fe LDH,^[^ ^28^ ^]^ and double‐shelled Ni–Fe LDH.^[^ ^29^ ^]^ Further, at extreme potentials (both anodic and cathodic), the current is restricted by the rate of diffusion of the electroactive species to the electrode surface. When the applied potential considerably speeds up the reaction, the bulk concentration of electroactive sites approaches zero. This results in a steady‐state diffusion‐limited current, with the peak current flattening into a sharp “shuttle” shape.^[^ ^30^ ^]^ The total charge contribution of the FSCs was calculated from the area under the CV curve.^[^ ^31^ ^]^ The surface morphology, crystalline properties, and chemical structure during different etching times led to the variation of contact resistance and hence the charge‐storing capacity of the electrode as shown in Figure S5 (Supporting Information).

Analysis of the CV results for various Ni‐Fe LDH samples (etched for 2, 4, and 8 h) reveal that specific capacitance decreases with longer etching times, as shown in Figure 4e. The NiFe‐LDH 2 h sample exhibits the highest specific capacitance of ≈9.24 mF·cm⁻^2^ at a scan rate of 2 mV·s⁻¹, leading to a maximum energy density of 0.46 µWh·cm⁻^2^, as depicted in Figure 4f. This drop in capacitance with increased etching time suggests that longer etching reduces the number of active sites available for charge storage due to changes in surface structure, as confirmed by FE‐SEM and TEM analyses. However, despite the decrease in specific capacitance with longer etching times, the 2 h etching sample maintains an optimal balance between redox and electrostatic contributions, making it the most promising candidate for energy storage applications. The XPS analysis indicates that the ratio of Ni^2+^: Ni^3+^ decreases with the increase in aging time. The ratios of Ni^2+^: Ni^3+^ and Fe: Ni in electrochemical systems, particularly in nickel‐iron (Ni‐Fe) oxides or hydroxides, considerably impact electrochemical reactions and performance. The Ni^2+^: Ni^3+^ ratio defines nickel‐containing materials' redox activity and electrical characteristics. The availability of Ni^3+^ is critical because it serves as an active site in electrochemical reactions. A more significant Ni^3+^ proportion increases the material's ability to engage in electron transport, which boosts activity. However, excessive Ni^3+^ concentrations might cause structural instability or the production of less conducting phases. The Ni^2+^ state is generally more structurally stable, although Ni^3+^ leads to increased conductivity because of its electronic structure. Excess Ni^3+^ can produce lattice deformation or phase transitions (e.g., from hydroxides to oxyhydroxides), which can reduce durability. An ideal balance is required to maintain both stability and performance.^[^ ^32^ ^]^ Iron inclusion causes a local structural disturbance, which can reveal additional active sites. It also influences the material's band structure, which improves electron transfer efficiency. A balanced Fe: Ni ratio ensures that the electrode material remains stable and active. Fe addition often enhances the corrosion resistance of nickel‐based products in alkaline environments. However, excessive Fe might cause phase segregation or decreased mechanical stability.^[^ ^33^ ^]^ The interplay of Ni^2+^: Ni^3+^ and Fe: Ni ratios have a direct impact on the activity, stability, and efficiency of electrochemical systems, making their optimization critical for advanced energy storage applications.

**Figure 5:**
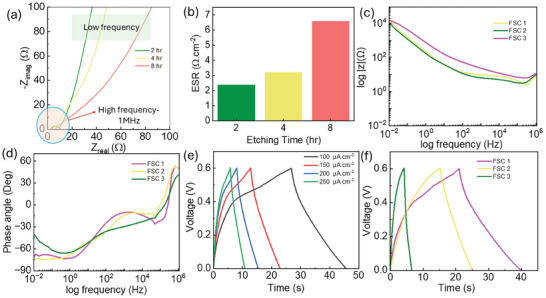
a) Nyquist plot reveals the resistance variation during electrode/electrolyte interaction of the SC developed on various etching times of NiFe‐LDH. b) ESR values for the FSCs. c) Bode impedance. d) Bode phase angle plot for the FSC1, FSC2 and FSC3. e) GCD curve for FSC1 at different current densities. f) Comparison of GCD curve three devices.

In addition to the pseudo‐capacitance behavior evaluation, the electrode/electrolyte interaction and ion kinetics were investigated through EIS analysis. The EIS test was conducted across a frequency range of 10–1 MHz for FSC1‐FSC3, as shown in the Nyquist plot in Figures S6a–c (Supporting Information) for individual FSCs. **Figure** **5**a,b illustrates the variation in the ESR value, revealing that the increase in ESR with prolonged etching time suggests structural and morphological changes in the electrode material, leading to higher electrolyte resistance. The Nyquist plot in Figure 5a indicates enhanced overall system resistance, with ESR values escalating from 2.4 Ω.cm^−2^ for the 2 h etching sample to 3.23 Ω. cm^−2^ for 4 h etching sample and 6.61 Ω. cm^−2^ for 8 h etching sample, as shown in Figure 5b in high‐frequency range. The electrode/electrolyte interaction shows that the SC made on an extended etching time sample (8 h) exhibits high resistance (as shown in Figure 5a) in the low‐frequency range compared to two other samples. On the other hand, the charge transfer resistance (R_ct_), represented by the diameter of the semicircle in the Nyquist plot (Figure 5a), decreases from 6.5 Ω·cm^2^ for FSC1 to 2.4 Ω·cm^2^ for FSC2 and 1.9 Ω·cm^2^ for FSC3. The lower R_ct_ of 1.9 Ω·cm^2^ for the 8 h etching sample indicates faster charge transfer kinetics at the electrode‐electrolyte interface. Further, we measured the changes in parameters through an equivalent fitting circuit for the FSC1‐FSC, as shown in Figure S7a–d (Supporting Information) and the observed variation of parameters given in Table TS4 (Supporting Information). The Bode plot in Figure 5c shows that the impedance of all three SCs increases as the frequency decreases, indicating capacitive behavior. The phase angles at lower frequencies are ≈−71.31° for FSC1, −72.88° for FSC2, and −39.6° for FSC3, as shown in Figure 5d, further indicating nearly capacitive behavior for these devices.

GCD studies were conducted on the devices using different current densities, as shown in Figure 5e for FSC1. Similar GCD curves for FSC2 and FSC3 are presented in Figure S8a,b (Supporting Information). The GCD curves for the FSCs deviate from the typical triangular shape expected of ideal SCs, indicating pseudocapacitive behavior in the devices. These curves also reveal that the developed SC samples exhibit long discharge times, particularly at the lowest current density of 100 µA·cm⁻^2^ across all samples, as illustrated in Figure 5f. The SC performance was specifically evaluated at this current density to achieve the maximum performance of the electrode materials. As the etching time increases, the discharge time decreases, indicating a decline in SC's performance. The maximum specific capacitance of 3.144 mF·cm⁻^2^ and a corresponding energy density of 0.157 µW·h·cm⁻^2^ were observed for FSC1 at the current density of 100 µA·cm⁻^2^. This suggests that the NiFe‐LDH sample with the shortest etching time (2 h) exhibits the best electrochemical performance among the tested samples. The measured energy and power densities of the FSCs were compared through the Ragone plot, as shown in Figure S9 (Supporting Information). When comparing these results with other SCs fabricated using similar LDHs, it becomes clear that the method of preparation, electrode properties, type of SC, and operating window significantly influence energy storage performance. For example, a hybrid supercapacitor utilizing Al‐doped NiCo LDH nanosheet arrays reported an energy density of 0.84 mWh.cm^−2^,^[^ ^34^ ^]^ while an Fe–Ni₃Co₂ LDH//FeSe₂/C device achieved an energy density of 22.3 µWh. cm^−^^2^.^[^ ^6^ ^]^ A NiCo‐LDH/NiCoOOH film demonstrated a higher energy density of 10.7 mWh. cm^−2^,^[^ ^35^ ^]^ and a P‐NiCoNW‐LDH/CC‐150//OCC asymmetric supercapacitor showed an energy density of 364 µWh. cm^−^^2^.^[^ ^36^ ^]^

**Figure 6:**
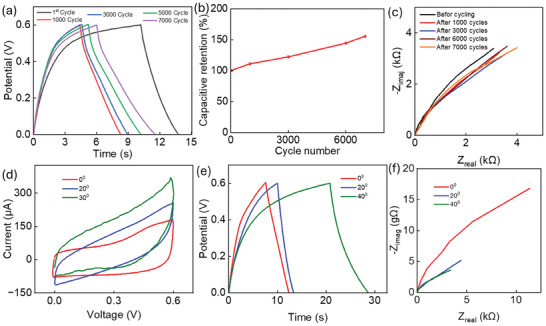
a) GCD curve for different cycles during long charging–discharging. b) Capacitive retention for long charging–discharging. c) Nyquist plot for the device in different intervals of long charging–discharging cycles. Flexibility performances of the devices under different bending angles. d) CV curve, e) GCD, and f) EIS analysis.

Cyclic stability is a crucial factor in determining the energy storage performance and efficiency of FSCs. It is typically expressed as specific capacitance or as capacitance retention (%). The cyclic stability of the FSC1 electrode in 6 m KOH electrolyte was evaluated by performing GCD measurements at a specific current of 200 µA·cm⁻^2^ over 7000 cycles within a voltage window of 0–0.6 V. The GCD curves for various cycle numbers are presented in **Figure** **6**a. The coulombic efficiency of the device was observed to vary with long charging‐discharging. For cycle 1 the efficiency is 35%, which is increased to 86% after 1000 cycles. After 7000 cycles the efficiency is increased to 97%. This could be due to the variation of the electrode properties during the cycling and is found in the structural variation of the material which is evaluated through FTIR analysis (given in Figure S10, Supporting Information). The intensity of the peaks varied after long charging discharging due to the reaction with electrolyte. Further after 7000 cycles, the specific capacitance of the electrode increased from 1.2 to 1.86 mF·cm⁻^2^, demonstrating a 150% capacity retention, as shown in Figure 6b. This improvement is attributed to the depletion of oxygenated functional groups on the electrode surface.^[^ ^37^ ^]^ Further in situ or operando analysis needs to investigate the understanding of the changing performance of the materials during long charging and discharging. Figure 6c analyses the EIS data at different cycling intervals, revealing an increase in overall impedance with continued cycling.

To evaluate the SC's flexibility, the device was bent at angles of 20° and 40°. CV (Figure 6d), GCD (Figure 6e), and EIS (Figure 6f) tests were conducted at these varying bending angles. The CV curves demonstrate that the capacitance increases with larger bending angles, as illustrated in Figure 6d. At a bending angle of 40°, the specific capacitance of the FSC increases by 1.6 times. This enhancement is attributed to the improved contact between the two electrodes and a reduction in resistance, as evidenced by the EIS analysis in Figure 6f. As the bendability increases, the improved contact between the electrodes reduces the device's overall resistance. These SCs are thus well‐suited for integration with flexible circuits, making them ideal for powering low‐power sensors and electronic components in wearable and smart devices.

## 3 Conclusions

In this work, we synthesized both double‐ and single‐shelled NiFe‐LDH particles using the octahedral spindle‐shaped MIL‐101(Fe) as a template. As the reaction time increased from 2 to 8 h, densely stacked sheets of NiFe‐LDH formed. This can be attributed to the fact that longer reaction and etching times facilitate greater etching and continuous metal ion deposition, resulting in thicker LDH shells on the MOF surface. Consequently, the as‐synthesized NiFe‐LDH particles transformed from double‐shelled to hollow single‐shelled structures as aging time increased. Based on these NiFe‐LDH powders, we fabricated three flexible supercapacitors (FSCs): FSC1 (NiFe‐LDH 2 h), FSC2 (NiFe‐LDH 4 h), and FSC3 (NiFe‐LDH 8 h). Electrochemical characterizations revealed that the samples with shorter etching times (2 h) exhibited superior energy storage performance due to their distinct morphological and structural properties. Specifically, the 2‐h etching time sample of NiFe‐LDH achieved the highest capacitance of 9.24 mF·cm⁻^2^ and an energy density of 0.46 µW·h·cm⁻^2^ at a scan rate of 2 mV·s⁻¹. The maximum specific capacitance of 3.144 mF·cm⁻^2^ and corresponding energy density of 0.157 µW·h·cm⁻^2^ were observed for FSC1 at a current density of 100 µA·cm⁻^2^. The excellent performance of this FSC, even under varying bending angles, underscores its potential for use in flexible devices and portable electronics.

## 4 Experimental Section

### Synthesis of MIL‐101 (Fe)

A one‐step solvothermal synthesis, followed by solvent exchange and vacuum activation was employed for the synthesis of MIL‐101 (Fe).^[^ ^18^, ^19^ ^]^ 5 mmol of FeCl_3_.6H_2_O was sonicated in 20 mL DMF, followed by the dropwise addition of 2.5 mmol H2BDC dissolved in 10 mL dimethylformamide (DMF). The mixture was then sonicated for another 1 h until it turned bright orange‐yellow. The reaction mixture was transferred into a stainless‐steel autoclave lined with Teflon and subjected to heating at 110 °C for 20 h. Following the cooling process, the resulting orange solid was collected via centrifugation. The product obtained was centrifuged and washed with DMF(x2) followed by ethanol heated at 60 °C (x3). The centrifuged sample was air‐dried at 60 °C for 3 h and vacuum‐activated at 150 °C for 24 h.

### Synthesis of NiFe‐LDH

A certain amount of the above synthesized MIL‐101(Fe) (≈0.1 g) was dispersed in a mixture of ethanol and deionized (DI) water in the ratio of 4: 6. Further, Ni (NO_3_)_2_.6H_2_O and urea (3: 2 wt. ratio) were added into the above mixture and aged in an oil bath at 110 °C. The reaction time was varied, and three different LDHs aged 2, 4, and 8 h were synthesized. After aging, the precipitate formed was centrifuged and washed multiple times with DI water and ethanol. A second etching step was adopted for the evolution of a double‐shelled structure. The washed solid was further dispersed in a 4:6 mixture of ethanol and water and allowed to be heated at 110 °C for 1 h with constant stirring. The obtained product was then washed several times with DI water and ethanol and dried under vacuum at 60 °C for 6 h.^[^ ^38^ ^]^ Correspondingly, the obtained samples were named as follows: NiFe‐LDH 2 h, NiFe‐LDH 4 h, and NiFe‐LDH 8 h.

### Supercapacitor Fabrication

Electrode fabrication for the FSC was commenced by utilizing a 1 cm^2^ flexible polyvinyl chloride (PVC) substrate. A graphene sheet (Graphene Supermarket, USA) acting as a current collector of the identical area was affixed to the PVC using insulating paste (JE solution) and placed into the oven for 15 min at 80 °C to get dry. External connectivity was established through a conducting wire soldered to the graphene electrode with the silver paste (RS component) by placing it in the oven for 30 min at 80 °C. In the subsequent step, a mixture of Ni‐Fe LDH, carbon black (Alfa Aesar‐ 45527), and binder, in an 8:1:1 ratio, was formulated with a 5 wt.% PVDF+DMF solution. This homogeneous slurry was then meticulously hand‐coated onto the electrodes and subjected to drying at 80 °C, ensuring solidification of the active material‐binder matrix. A cellulose paper separator was positioned between the electrodes to prevent electrical short circuits while enabling ion migration during electrochemical reactions. Two drops of KOH solution were carefully deposited onto the separator to serve as the electrolyte. Finally, the entire assembly was encased in cling film for insulation and protection against environmental factors.

### Characterization

XRD analysis confirmed the MOF to the corresponding NiFe LDH. The examination utilized a Siemens D500 X‐ray powder diffractometer, operating with Cu Kα radiation (λ = 0.15406 nm) at 40 kV and 30 mA. The diffraction pattern was captured over a range spanning 10°–80°, with a step size of 0.2° per increment and a dwell time of 30 s per step. Surface morphology examination was conducted using FE‐SEM (Zeiss Gemini Ultra) equipped with an In Lens detector, operating at an acceleration voltage of 2 kV and a working distance of ≈3.7 mm. TEM was performed using a JEOL LaB6 gun at 200 kV. Image analysis was carried out using ImageJ software to explore alterations in particle sizes.FTIR was employed with a Perkin Elmer spectrometer (Spectrum 100) and an MCT detector within the 400–4000 cm^−1^ range to analyze the chemical bonds within the samples. KBr discs were used as the background, and the powder samples were mixed with KBr powder to make discs, which were further analyzed using FTIR. Raman spectroscopy (Horiba Jobin Yvan Lab RAM HR 800) was used to study phase transformations with a 600 gr mm^−1^ grating. A solid‐state diode laser with a wavelength of 532 nm was used to analyze the powder samples, with an acquisition time of 150 s and accumulating 3 scans per measurement. XPS was utilized to explore the chemical composition, oxidation states of the elements, and bonding interactions. The XPS measurements were conducted using a Thermo Scientific K‐Alpha+ spectrometer with a monochromatic Al Kα X‐ray source (hν = 1486.6 eV). The survey spectra were obtained through five scans with a dwell time of 10 ms and a step size of 0.4 eV, while the high‐resolution spectra were acquired through ten scans with a dwell time of 50 ms and a step size of 0.1 eV. The survey spectra used a pass energy of 200 eV, whereas the high‐resolution core‐level spectra for each element utilized a pass energy of 50 eV. All spectra were calibrated relative to the C1 s peak at 285 eV to mitigate the charging effects during data collection. The XPS data was analyzed using CasaXPS version 2.3.25PR1.0, applying a Voight‐style line shape containing a mixture of Gaussian and Lorentzian components in a 65:35 ratio correction for all core‐level spectra.

The electrochemical characterization includes CV (2–1000 mV.s^−1^ at a voltage window ranging from 0 to 0.6 V), EIS (10 mHz to 1 MHz at a potential of 10 mV), and GCD (various current densities) was carried out by using electrochemical workstation IviumStat2.h‐ (Ivium Technologies B.V). The wettability of the SC is measured by contact angle equipment (Ossila Contact Angle v3.1.2.2). Conductivity is measured by four probe methods (Ossila Sheet Resistance Lite v1.0.4.1)

## Conflict of Interest

The authors declare no conflict of interest.

## Supporting information



Supporting Information

## Data Availability

Data sharing is not applicable to this article as no new data were created or analyzed in this study.
