# Supplementary material for: Spindle‐Shaped Ni‐Fe‐Layered Double Hydroxide: Effect of Etching Time on Flexible Energy Storage
Source: Small. 2025 Jan 10;21(7):2409959. doi: 10.1002/smll.202409959 (PMC11840465; doi:10.1002/smll.202409959)
Supplement: Supplementary file 1 — Supporting Information [file SMLL-21-2409959-s001.docx]

Supporting Information

Spindle-Shaped Ni-Fe-Layered Double Hydroxide: Effect of Etching Time on Flexible Energy Storage

Keerthi M. Nair, Sindhya Ajith, Febin Paul, Sreedhanya Pallilavalappil, Nishanth Thomas, Steven J. Hinder, Libu Manjakkal, Suresh C. Pillai*

Table TS1. The summarized glance of the particle size of MIL-101(Fe) as observed from FE-SEM.

| Particles | Length | Equatorial Diameter |
| --- | --- | --- |
| 1 | 488.89 | 300.00 |
| 2 | 491.16 | 290.81 |
| 3 | 600.10 | 222.52 |
| 4 | 480.87 | 268.97 |
| 5 | 578.21 | 233.33 |
| 6 | 533.45 | 200.00 |
| Mean | **528.78** | **252.60** |

Table TS2. Summarized glance of the distance between the inner and outer shells of the as-synthesized samples. The table indicates that the central void space starts increasing with the prolonged reaction time, eventually leading to the formation of a complete hollow structure, Ni-Fe LDH 8h.

| Sample | Distance between the inner and outer shells | Mean Distance |
| --- | --- | --- |
| Ni-Fe LDH 2hr | 52.459  41.731  41.634 | 45.27466667 |
| Ni-Fe LDH 4hr | 85.78  73.995  118.238 | 92.671 |
| Ni-Fe LDH 8hr | ----- | ------ |


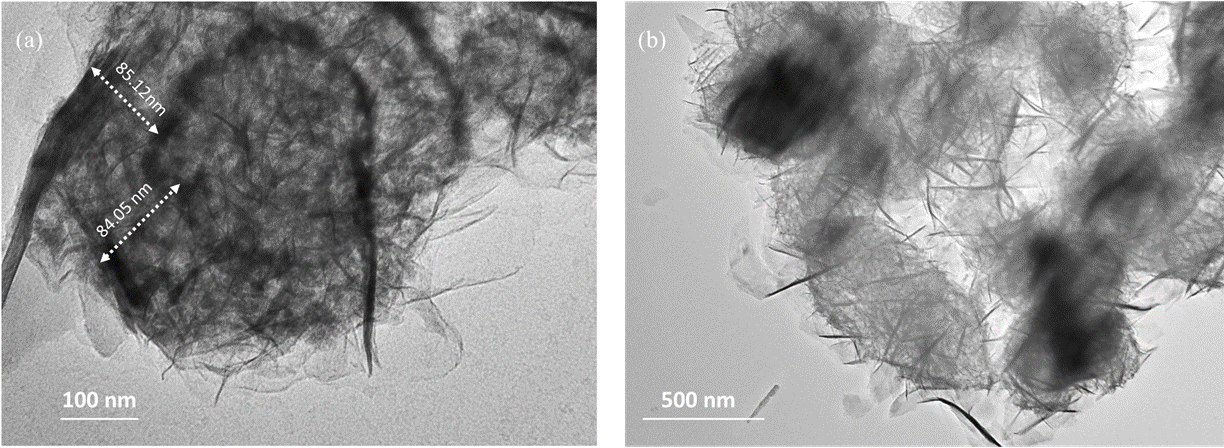


Figure S1: TEM images of (a) Ni-Fe LDH 4h, showing a breakage of the inner shell indicating a longer reaction time could destroy the double shelled structure leading to the formation of a hollow LDH. (b) NI-Fe LDH 8h, with hollow spindle structures, the higher number of flakes which lead to dense aggregation resulting in the formation of an extremely interconnected structure.


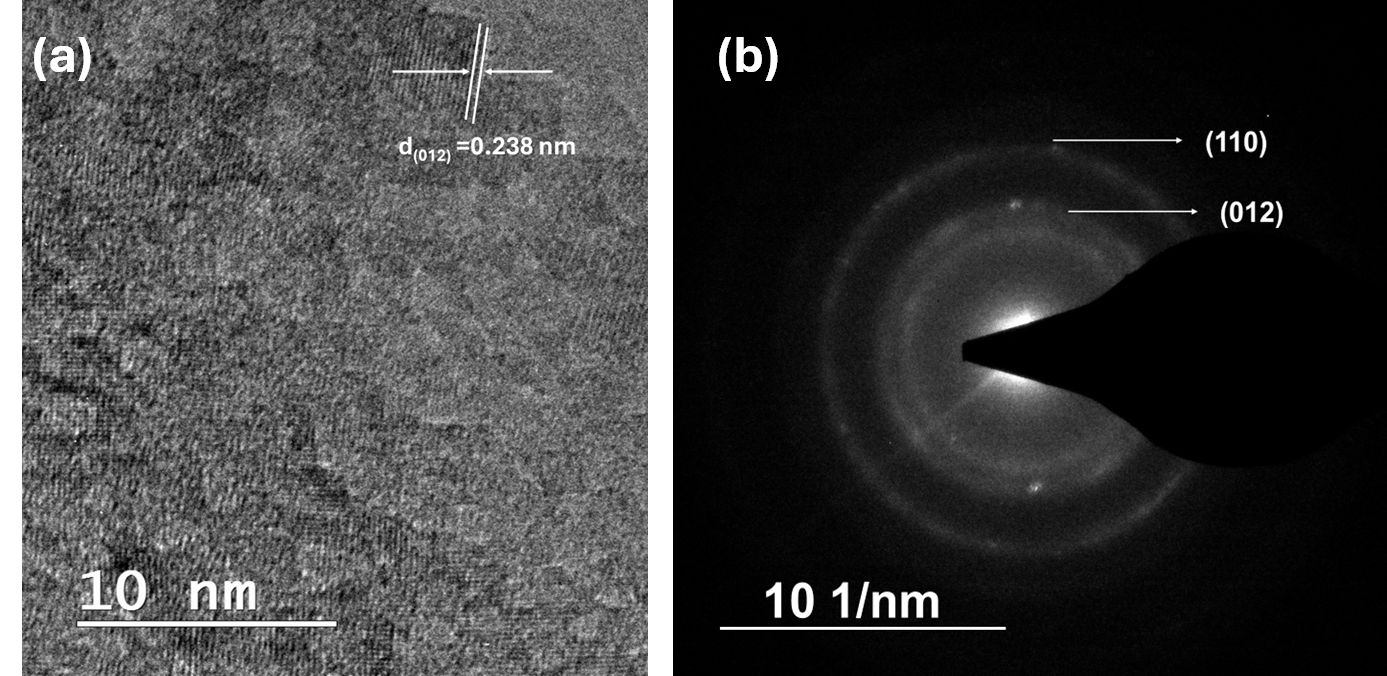


Figure S2: HRTEM images of (a) Ni-Fe LDH 2h, showing the d-spacing of 0.238 nm (b) SAED pattern of NiFe LDH-2h.

Table TS3. Summarized glance of the interlayer distance and crystal size of the as-synthesized NiFe-LDH.

| Parameters​ | 2h​ | 4h​ | 8h​ |
| --- | --- | --- | --- |
| d_003_ (nm)​ | 0.71​ | 0.72​ | 0.70​ |
| d_012_ (nm) | 0.25 | 0.26 | 0.26 |
| Crystallite size in the c direction (nm)​ | 11.2​ | 10.7​ | 11.5​ |


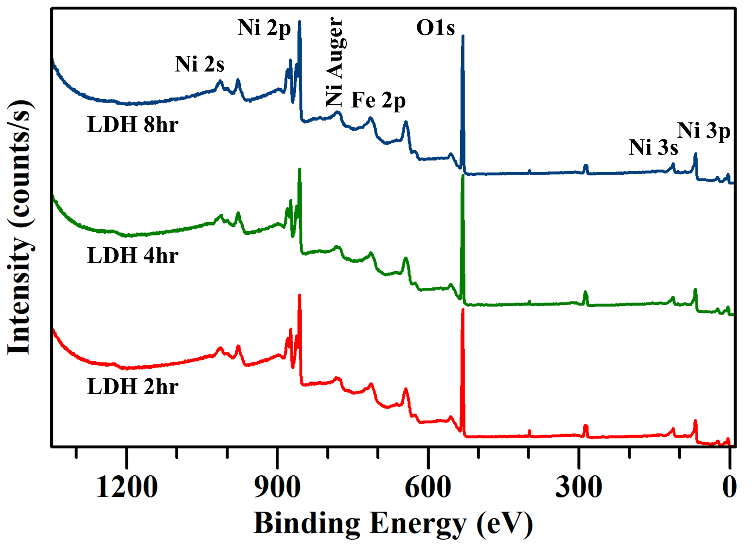


Figure S3: Survey spectra of all three LDH samples, NiFe-LDH-2hr, NiFe-LDH-4hr, and NiFe-LDH-8hr.


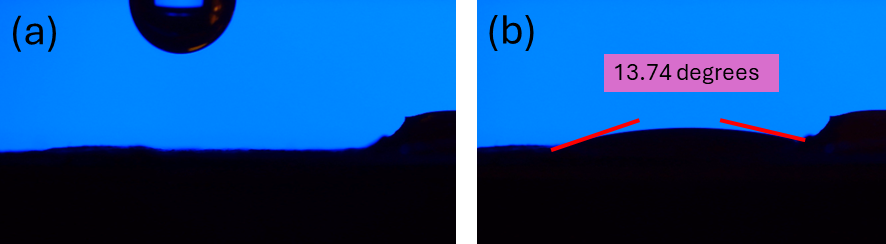


Figure S4: Contact angle of NiFe-LDH 2h a) immediately after dropping (b) after 30 seconds


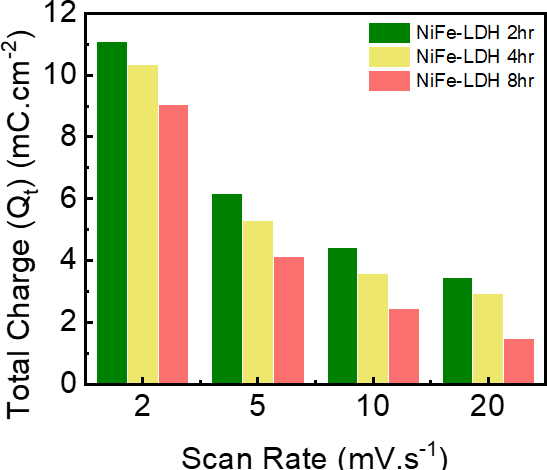


Figure S5: Total charge contribution in each FSCs.


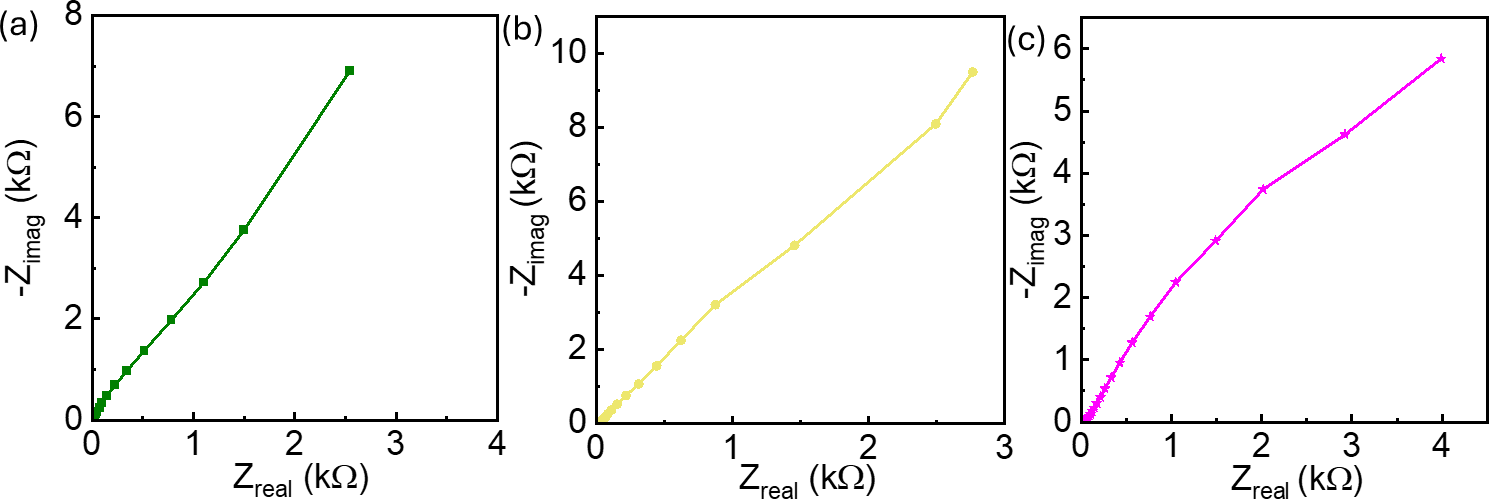


Figure S6: Nyquist plots for (a) FSC1 (NiFe-LDH-2hr) (b) FSC2 (NiFe-LDH- 4hr (c) FSC3 (NiFe-LDH- 8hr)


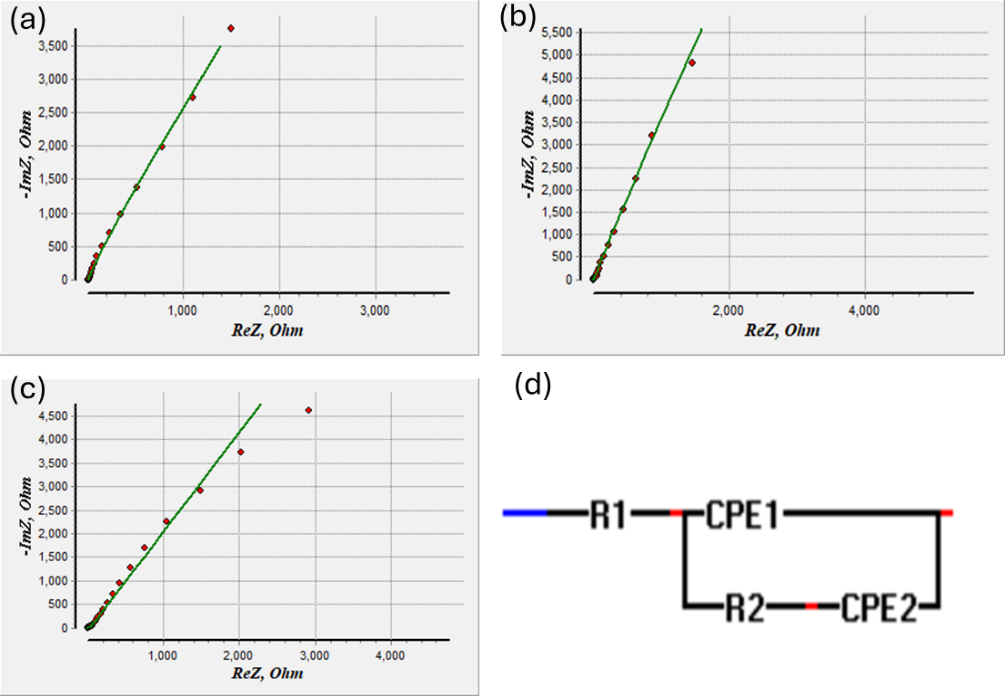


Figure S7: Equivalent circuit fitting for (a) FEC1 (b) FSC2 and (c) FSC3. (d) the circuit used for fitting.

Table TS4: Equivalent circuit fitted parameters of the FSCs (R1 represent the ESR and R2 represents the bulk resistance)

|  | FSC1 | FSC2 | FSC3 |
| --- | --- | --- | --- |
| R1(Ω) | 3.5822 (41.97%) | 3.8045 (35.33%) | 6.545 (15.11%) |
| R2 (Ω) | 8.0879 (81.87%) | 14.917 (195.32%) | 43.382 (42.89%) |
| P1 (F⋅s^n−1^) | 0.00049 (75.46%) | 0.000499 (86.36%) | 0.0001 (25.12%) |
| n1 | 0.65958 (13.57%) | 0.758 (8.51%) | 0.674 (4.11%) |
| P2 (F⋅s^n−1^) | 0.0005 (59.74%) | 0.0005 (122.63%) | 0.00025 (18.63%) |
| n2 | 0.9162 (10.88%) | 0.9153 (12.26%) | 0.734 (6.91%) |


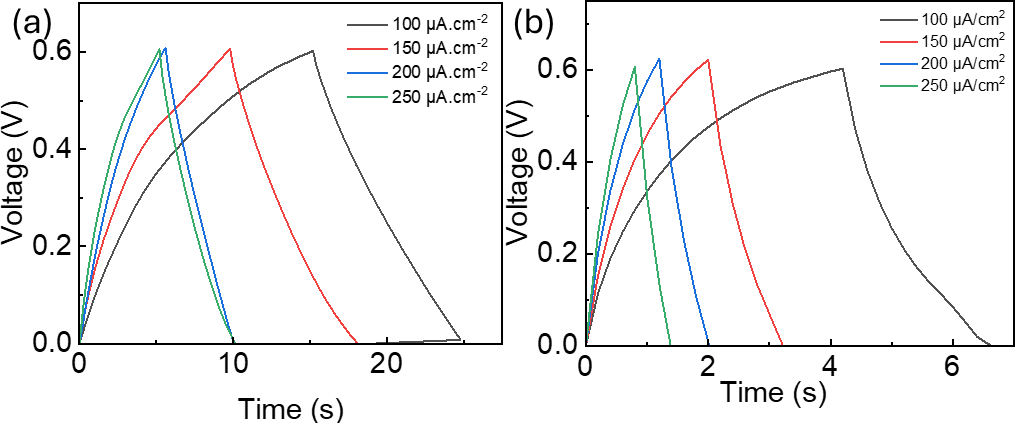


Figure S8: GCD plots for FSC2 (NiFe-LDH- 4hr (b) FSC3 (NiFe- LDH- 8 hr)


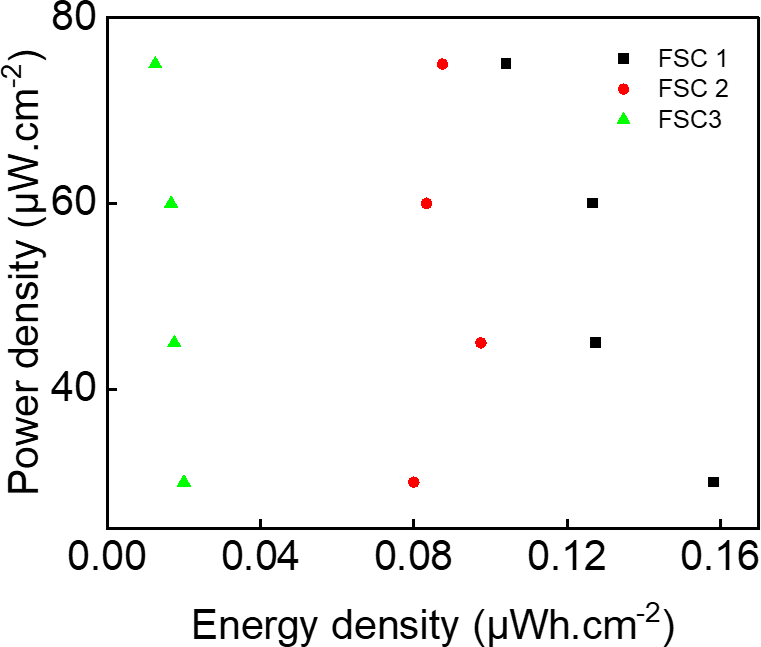


Figure S9: Ragone plots for the FSCs


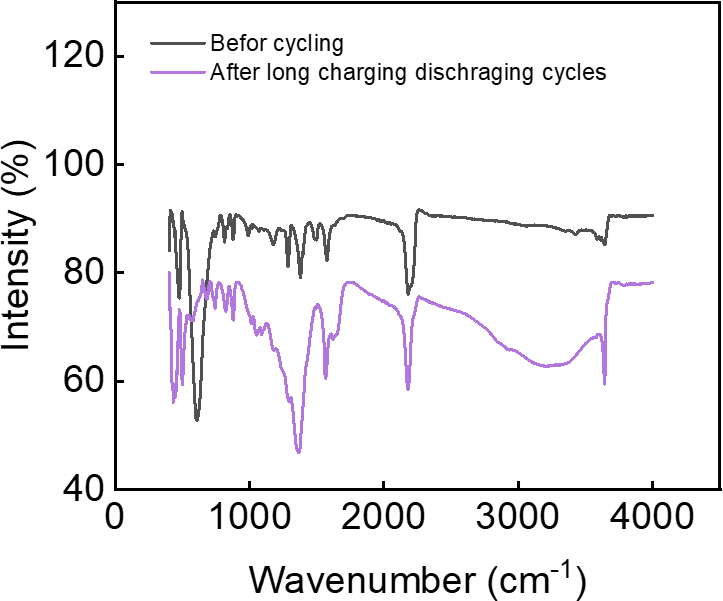


Figure S10: FTIR spectra of NiFe-LDH before and after long charging discharging cycles.
